# Mucorales-Specific T Cells in Patients with Hematologic Malignancies

**DOI:** 10.1371/journal.pone.0149108

**Published:** 2016-02-12

**Authors:** Leonardo Potenza, Daniela Vallerini, Patrizia Barozzi, Giovanni Riva, Andrea Gilioli, Fabio Forghieri, Anna Candoni, Simone Cesaro, Chiara Quadrelli, Johan Maertens, Giulio Rossi, Monica Morselli, Mauro Codeluppi, Cristina Mussini, Elisabetta Colaci, Andrea Messerotti, Ambra Paolini, Monica Maccaferri, Valeria Fantuzzi, Cinzia Del Giovane, Alessandro Stefani, Uliano Morandi, Rossana Maffei, Roberto Marasca, Franco Narni, Renato Fanin, Patrizia Comoli, Luigina Romani, Anne Beauvais, Pier Luigi Viale, Jean Paul Latgè, Russell E. Lewis, Mario Luppi

**Affiliations:** 1 Section of Hematology, Department of Surgical and Medical Sciences, University of Modena and Reggio Emilia, Azienda Ospedaliero-Universitaria Policlinico, Modena, Italy; 2 Hematology and Bone Marrow Transplantation, Azienda Ospedaliero-Universitaria Santa Maria della Misericordia di Udine, Udine, Italy; 3 Pediatric Hematology/Oncology, Policlinico GB Rossi, Verona, Italy; 4 Department of Hematology, Universitaire Ziekenhuizen Leuven, Campus Gasthuisberg, Leuven, Belgium; 5 Department of Pathology, Azienda Ospedaliero-Universitaria Policlinico, Modena, Italy; 6 Clinic of Infectious Diseases, Integrated Department of Medicine, Emergency Medicine and Medical Specialties, Azienda Ospedaliero-Universitaria Policlinico, Modena, Italy; 7 Section of Statistics, Department of Diagnostic, Clinical and Public Health Medicine, University of Modena and Reggio Emilia, Azienda Ospedaliero-Universitaria Policlinico, Modena, Italy; 8 Division of Thoracic Surgery, Department of Surgical and Medical Sciences, University of Modena and Reggio Emilia, Azienda Ospedaliero-Universitaria Policlinico, Modena, Italy; 9 Pediatric Hematology/Oncology and Transplantation, IRCCS Policlinico S. Matteo, Pavia, Italy; 10 Department of Experimental Medicine and Biochemical Sciences, University of Perugia, Perugia, Italy; 11 Unitè des Aspergillus, Pasteur Institut, Paris, France; 12 Clinic of Infectious Diseases, Department of Internal Medicine, Geriatrics and Nephrologic Diseases, S. Orsola-Malpighi Hospital, University of Bologna, Bologna, Italy; University of Szeged, HUNGARY

## Abstract

**Background:**

Invasive mucormycosis (IM) is an emerging life-threatening fungal infection. It is difficult to obtain a definite diagnosis and to initiate timely intervention. Mucorales-specific T cells occur during the course of IM and are involved in the clearance of the infection. We have evaluated the feasibility of detecting Mucorales-specific T cells in hematological patients at risk for IM, and have correlated the detection of such cells with the clinical conditions of the patients.

**Methods and Findings:**

By using an enzyme linked immunospot assay, the presence of Mucorales-specific T cells in peripheral blood (PB) samples has been investigated at three time points during high-dose chemotherapy for hematologic malignancies. Mucorales-specific T cells producing interferon-γ, interleukin-10 and interleukin-4 were analysed in order to detect a correlation between the immune response and the clinical picture. Twenty-one (10.3%) of 204 patients, accounting for 32 (5.3%) of 598 PB samples, tested positive for Mucorales-specific T cells. Two groups could be identified. Group 1, including 15 patients without signs or symptoms of invasive fungal diseases (IFD), showed a predominance of Mucorales-specific T cells producing interferon-gamma. Group 2 included 6 patients with a clinical picture consistent with invasive fungal disease (IFD): 2 cases of proven IM and 4 cases of possible IFD. The proven patients had significantly higher number of Mucorales-specific T cells producing interleukin-10 and interleukin-4 and higher rates of positive samples by using derived diagnostic cut-offs when compared with the 15 patients without IFD.

**Conclusions:**

Mucorales-specific T cells can be detected and monitored in patients with hematologic malignancies at risk for IM. Mucorales-specific T cells polarized to the production of T helper type 2 cytokines are associated with proven IM and may be evaluated as a surrogate diagnostic marker for IM.

## Introduction

Invasive mucormycosis (IM) is a severe fungal infection that affects patients with various clinical conditions, all characterized by some degree of impaired immune response, including diabetes, iron overload, organ transplantation and hematologic malignancies [1–6]. The crude mortality rate of IM is as high as 70%, a number that has not decreased over recent years [6–12]. The non-specific clinical presentation of IM is certainly one of the main reasons for this overall poor outcome. Indeed, from a clinical and radiological point of view, IM is indistinguishable from other infections, either fungal or bacterial [1–12]. Thus, a definite diagnosis of IM requires the demonstration of suggestive fungal hyphae in infected tissue together with a positive culture from that tissue. No alternative diagnostic methods exist. However, tissue biopsies are only rarely performed in patients at high risk for IM, due to their coagulopathy/low platelets [5, 10–17].

We and others have previously reported on the presence of specific T cells in patients with invasive infections caused by filamentous fungi [18–22]. These T cells contribute to human immune responses against these fungi, and seem to correlate with the outcome of the infection [19–22]. Specifically, we demonstrated that *Mucorales*-specific T cells could be detected in three patients with documented IM during the course of the infection. Such T cells could not be demonstrated before the infection or at complete resolution, and were completely absent in 25 controls without IM [20]. These findings suggest that the presence of *Mucorales*-specific T cells is strongly associated with the occurrence of IM.

In the present study, we have prospectively evaluated the feasibility of monitoring the presence of *Mucorales*-specific T cells in patients with hematologic malignancies undergoing treatment that puts them at high risk for invasive fungal diseases (IFD). Furthermore, we have investigated whether the presence of *Mucorales*-specific T cells correlates with specific clinical presentation of disease.

## Results

A total of 204 patients were enrolled into the study. The median age was 51.8 years (range 17–78). One hundred and four patients (51%) had acute myeloid leukemia (AML); 10 (5%) high-risk myelodysplastic syndrome; 28 (13%) acute lymphoblastic leukemia (ALL); 3 (2%) severe aplastic anemia, 15 (7%) non-Hodgkin lymphoma, and 44 patients (22%) underwent allogeneic hematopoietic stem cell transplant (alloSCT). Clinical characteristics of the patients are reported in Table 1.

**Table 1:** Clinical characteristics of the patients.

*Patients*	*204*
Median Age (yrs; min-max)	51.85 (15–78)
*Hematologic Malignancies*	204 (100)
Acute Myeloid Leukemia (%)	104 (51)
Acute Lymphoblastic Leukemia (%)	28 (13)
High-risk MDS (%)	10 (5)
Severe Aplastic Anemia (%)	3 (2)
Non-Hodgkin Lymphoma (%)	15 (7)
*AlloSCT*	44 (22)
sibling / haploidentical / MUD (%)	33 (75) / 6 (14) / 5 (11)
Anti-fungal Prophylaxis (%)	171 (84)
Posaconazole/Itraconazole/Fluconazole	66/41/64
*Febrile Episodes (%)*	127 (62)
Bacterial / Viral / Other (%)	25 (12) / 8 (4) / 5(1)
Proven or probable IA (%)	33 (16)
Proven IM (%)	2 (1)
Undetermined (Febrile Neutropenia / possible IFD) (%)	34 (17) / 20 (10)
BAL (%)	104 (51%)
*GM Samples*	
GM serum (tot/pos)	1557/77
GM BAL (tot/pos)	104/21
*ELISpot Samples*	598
Leukopenic samples at T1 (%)	182 /199 (91.5)
Leukopenic samples at T2 (%)	168 /203 (83)
Leukopenic samples at T3 (%)	73 /196 (37)

MDS = Myelodisplastic Syndrome; alloSCT = Hematopoietic Stem Cell Transplant; MUD = Matched Unrelated Donor; IA = Invasive Aspergillosis; IM = Invasive Mucormycosis; IFD = Invasive Fungal Disease; GM = galactomannan. Anti-fungal prophylaxis was performed with posaconazole in acute myeloid leukemia/MDS patients younger than 65 year-old and alloSCT patients with chronic graft versus host disease; with fluconazole in alloSCT patients; with itraconazole in acute myeloid leukemia/MDS patients older than 65 year-old.

**Fig 1:**
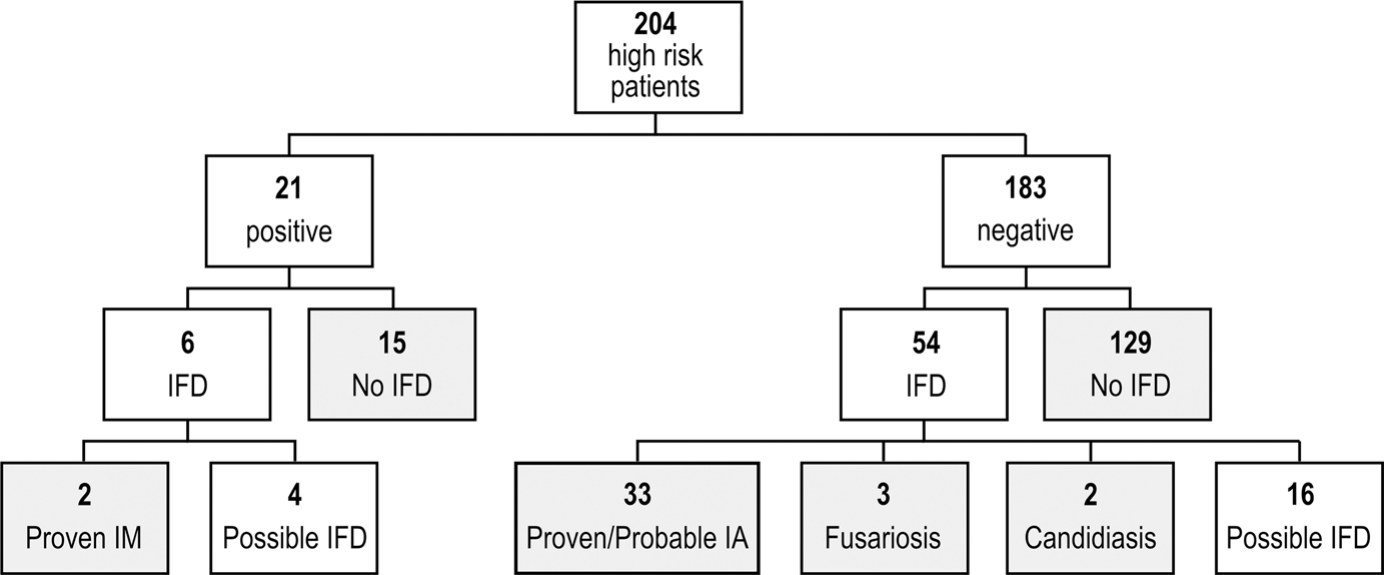
Classification of the patients according with the results of the ELISpot assay. IFD = invasive fungal disease. IM = invasive mucormycosis; IA = invasive aspergillosis. Grey boxes indicate the patients with a defined diagnosis considered for the computation of sensitivity, specificity and receiver-operating characteristic analysis.

According to the revised European Organization for Research and Treatment of Cancer and Mycoses Study Group (EORTC-MSG) criteria [23], 40 (19%) cases of invasive fungal disease (IFD) were diagnosed during the study period, including 33 (16%) cases of proven or probable invasive aspergillosis (IA), 2 (1%) cases of proven IM, 3 (1.5%) cases of proven fusariosis and 2 (1%) cases of invasive candidiasis. Furthermore, 20 patients were classified as having possible IFD (Fig 1).

A total of 598 peripheral blood (PB) samples were analyzed (average 2.93 per patient; range from 1 to 7). In order to study *Mucorales*-specific T cells producing interferon-gamma (IFN-γ), interleukin 10 (IL-10) and IL-4, 3 analyses were performed for each sample, resulting in a total of 1794 test results. Peripheral blood samples were obtained at the following time points [median day (minimum–maximum)]: during high dose induction chemotherapy/ immunosuppressive treatment: T1 = +16 (12–45); T2 = +32 (23–60); T3 = +46 (27–75); during alloSCT conditioning, T1 = +37 (12–117); T2 = +74 (20–197); T3 = +101 (40–180).

**Fig 2:**
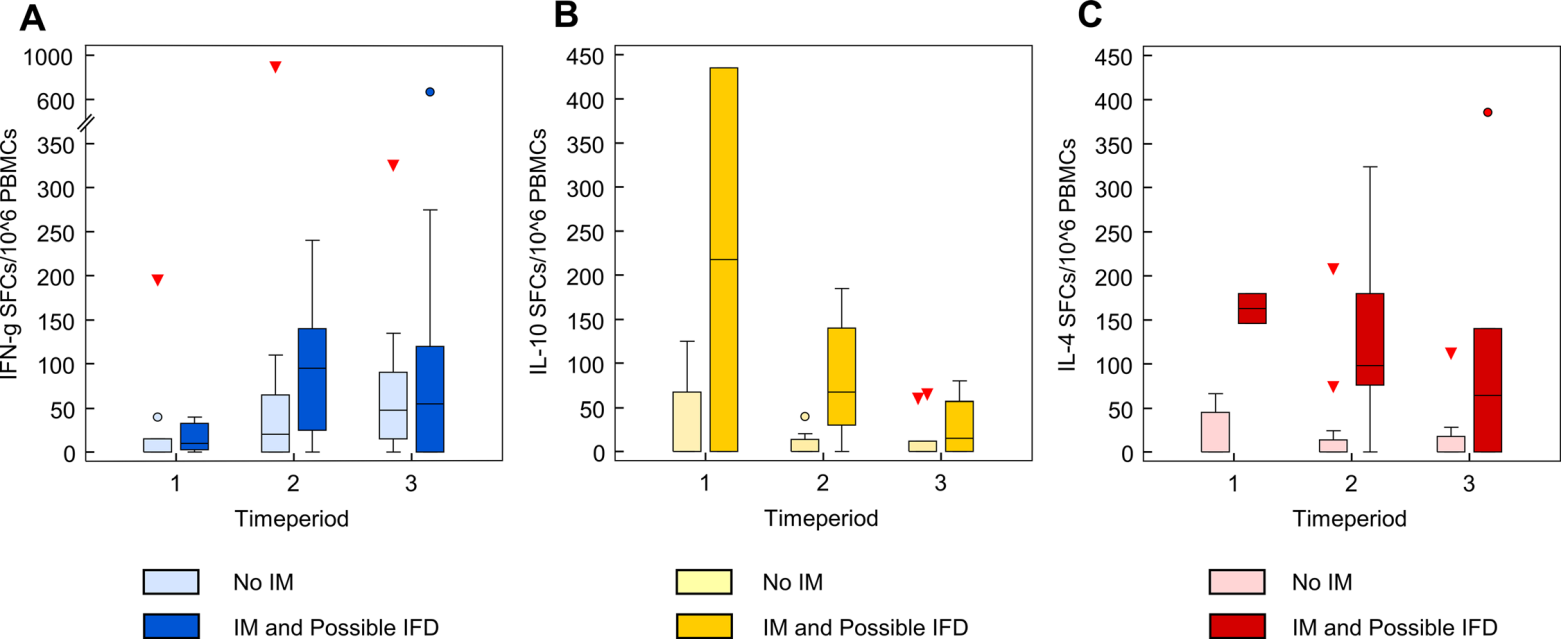
**(A-C). Kinetics of T-cell responses in the patients showing *Mucorales*-specific T cells.** (A) Kinetics of *Mucorales*-specific T cells producing IFN-γ (blue); (B) IL-10 (yellow); (C) IL-4 (red) in the 15 patients without IFD (light colours) and in the 6 patients with IFD (dark colours), 2 proven IM and 4 possible IFD, at each time period of the study. The central box represents the values from the lower to upper quartile (25 to 75 percentile). The middle line represents the median. The horizontal line extends from the minimum to the maximum value. Triangles and dots show "outside" and "far out" values, respectively. The vertical axis shows the number of spot-forming cells (SFCs) per million PBMCs producing a specific cytokine. The horizontal axis represents the three time periods, when peripheral blood samples have been obtained.

The ELISpot assay showed the presence of *Mucorales*-specific T cells in 21 of 204 patients (10.3%) (Figs 1 and 2). Clinical characteristics of these patients are summarized in Table 2. Such patients accounted for all the positive samples. Thirty-two (5.3%) samples tested positive: 5 at T1, 13 at T2, 14 at T3.

**Table 2:** Clinical characteristics of the 21 patients positive for *Mucorales*-specific T cells.

Pt n°	Sex	Age (yrs)	Condi-tion	Definite Diagnosis	HRCT appearence	Day of fever	Day of CT findings	Samples			
								n°	n° pos. (days)	n° neg. (days)	n° uninf.
1	F	57	AML	MRSA Pneumonia	Patchy consolidation with air bronchogram	+18	+22	3	2 (+33;+40)	1 (+20)	0
2	M	68	NHL	S. aureus Pneumonia	Bilateral interstitial pneumonia	+7	+7	2	2 (+13; +20)	0	0
3	M	22	B-ALL	No infection	No scan performed	n.a.	n.a.	3	2	1	0
4	M	26	AML	No infection	No scan performed	n.a.	n.a.	3	1	2	0
5	F	35	alloSCT	No infection	No scan performed	n.a.	n.a.	3	1	0	2
6	M	26	alloSCT	No infection	No scan performed	n.a.	n.a.	3	1	2	0
7	M	71	AML	Pneumonia resolved with antibiotics	Patchy consolidation with air bronchogram in right lung	+28	+31	3	1 (+46)	2 (+15; +29)	0
8	F	63	AML	Febrile neutropenia resolved with antibiotics	Hypoventilation in lower lobes	+26	+33	3	2 (+25; +40)	1 (+56)	0
9	F	65	AML	Pneumonia solved with antibiotics	Two areas of patchy consolidation in right lung	+31	+35	3	1 (+42)	2 (+15; +23)	0
10	F	68	AML	Interstitial pneumonia solved with antibiotics	Bilateral interstitial lung disease	+12	+16	2	2 (+21; +29)	0	0
11	M	27	AML	No Infection	No scan performed	n.a.	n.a.	3	1	2	0
12	M	50	B-ALL	No Infection	No scan performed	n.a.	n.a.	2	1	0	1
13	F	50	alloSCT	No Infection	No scan performed	n.a.	n.a.	1	1	0	0
14	F	52	alloSCT	No Infection	No scan performed	n.a.	n.a.	3	1	2	0
15	M	60	alloSCT	Febrile neutropenia rapidly solved with antibiotics	No scan performed	n.a.	n.a.	3	2	0	1
16	F	39	AML	Possible IFD	Left lung nodular lesion with air crescent sign	+20	+21	3	2 (+25; +38)	0	0
17	F	57	AML	Possible IFD	Nodular lesion in the left lung	+34	+39	2	1 (+23)	1 (+57)	0
18	M	50	MDS	Possible IFD	Multiple bilateral nodular areas with ground-glass opacifications, bilateral pleural effusions	+19	+21	3	2 (+21; +47)	1 (+34)	0
19	M	72	AML	Possible IFD	Large nodular lesion in the left lung surrounded by ground-glass opacities	+21	+24	3	2 (+16; +29)	1 (+ 42)	0
20	M	52	NHL	Proven IM	Soft tissue obliteration of paranasal sinuses and bone erosion	+25	+35	2	2 (+45; +62)	0	0
21	M	17	NHL	Proven IM	Large left pulmonary consolidation with central area of ground glass opacity	+20	+25	2	2 (+48; +55)	0	0

Pos. = positive; uninf. = uninformative; HRCT = High-Resolution Computed-scan Tomography; AML = acute myeloid leukemia; NHL = non-Hodgkin lymphoma; B-ALL = B-cell acute lymphoblastic leukemia; alloSCT = allogeneic hematopietic stem cell transplantation; MDS = myelodisplastic syndrome; MRSA = Methicillin-Resistant Staphylococcus Aureus; S. aureus = Staphylococcus aureus; IFD = Invasive Fungal Disease; n.a. = not applicable.

The median number [spot forming cells (SFCs) / 10^6^ peripheral blood mononuclear cells (PBMCs) with their 25th and 75th percentiles] of *Mucorales*-specific T cells producing IFN-γ was: 65 (40–127.5); those producing IL-10: 20 (20–65) and those producing IL-4: 60 (20–114), respectively (Fig 2).

**Fig 3:**
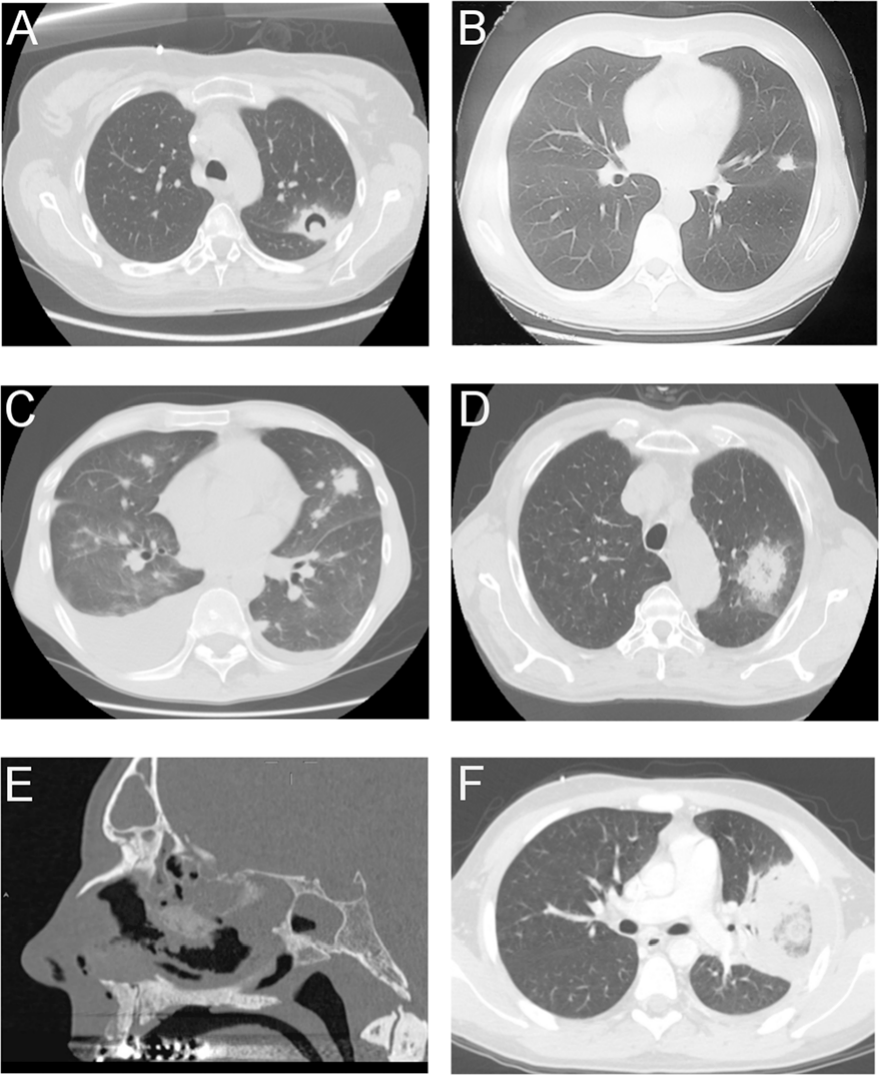
**(A-F). Radiologic findings of patients from group 2 with possible IFD (A–D) and proven IM (E and F).** (A) *Patient 16*. Well shaped nodular lesion of 4.5 cm, with surrounding area of ground glass, and air crescent sign in the posterior part of the upper left pulmonary lobe at HRCT. (B) *Patient 17*. Pulmonary HRCT demonstrating a small well shaped nodular lesion of the left upper lobe. (C) *Patient 18*. Pulmonary HRCT showing multiple bilateral well shaped nodular lesions, sometimes surrounded by ground-glass attenuation. Bilateral pleural effusions. (D) *Patient 19*. HRCT showing a large (4.7 cm) nodular lesion surrounded by an area of ground glass attenuation in the posterior part of the upper left pulmonary lobe. (E) *Patient 20*. Sagittal sinus CT scan. Soft tissue obliteration of ethmoid and middle meatus, erosion of ethmoid bony lamellae and spheno-etmoidal floor. Obliteration of frontal and sphenoidal sinus. (F) *Patient 21*. CT of the chest showing a central consolidation surrounded by a rim of ground-glass opacity within a large left hilar pulmonary consolidation (reverse halo-sign appearance). HRCT = high resolution computed tomography.

When we correlated the occurrence of *Mucorales*-specific T cells with the clinical outcome, patients with positive results could be grouped into two categories (Figs 1 and 2). The first group consisted of 15 patients (n = 1–15) with clinical, microbiological and radiological features unlikely to be related to IFD, because they met one of the following criteria: 1) they were diagnosed with proven non-fungal infections; 2) they never developed fever during the study period; 3) their infection resolved without the administration of antifungal agents (Table 2). The second group was composed of 6 patients with proven IM (n = 2) and possible IFD (n = 4), according to the EORTC/MSG criteria (Table 2 and Fig 3) [23].

**Fig 4:**
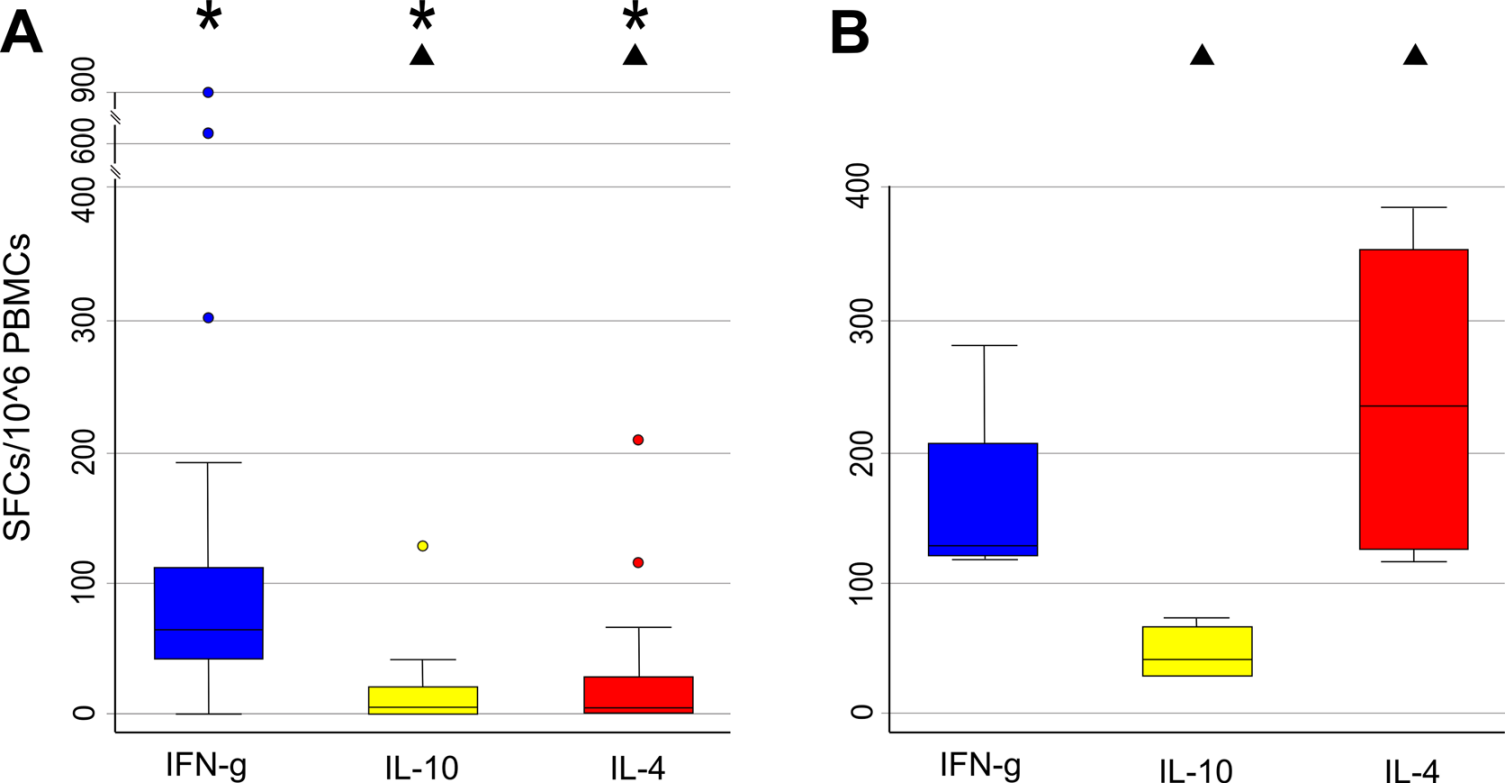
**(A,B) Comparison between frequencies of *Mucorales*-specific T cells in patients without IFD and with proven IM.** (A). Box plots showing specific immune responses producing IFN-γ (blue column), IL-10 (yellow column) and IL-4 (red column) in Group 1 patients (n = 15) without IFD. (B). Box plots showing specific immune responses producing IFN-γ (blue column), IL-10 (yellow column) and IL-4 (red column) in the 2 patients with proven IM. The vertical axis shows the number of spot-forming cells (SFCs) per million PBMCs. The horizontal axis represents the cytokine produced by *Mucorales*-specific T cells. The upper horizontal line represents the upper adjacent value. The upper hinge of the boxes represents the 75th percentile. The middle horizontal line of the boxes represents the median value. The lower hinge of the boxes represents the 25th percentile. The lower horizontal line represents the lower adjacent value. Blue, yellow and red dots are outrange values. * and ^▲^ = P< 0.05.

In the first group, the median number (SFCs/10^6^ PBMCs with their 25th and 75th percentiles) of *Mucorales*-specific T cells producing IFN-γ was 62.5 (40–115); those T cells producing IL-10 was 0 (0–20) and those producing IL-4 was 0 (0–28) as well. In this group of patients the differences in the number of *Mucorales*-specific T cells producing IFN-γ and IL-10, and IFN-γ and IL-4 resulted significantly different (p = 0.001) (Fig 4A). These findings are indicative of *Mucorales*-specific immune responses polarized to the production of IFN-γ in this group.

The second group was additionally split in the two patients with proven IM and in the four patients with possible IFD. In the 2 patients with proven IM, the median number (SFCs/10^6^ PBMCs with their 25th and 75th percentiles) of *Mucorales*-specific T cells producing IFN-γ was 132.5 (122.5–207.5); those T cells producing IL-10 was 43.5 (30–66); those T cells producing IL-4 was 232 (128–355). In these two patients only the difference between *Mucorales*-specific T cells producing IFN-γ- and IL-10 resulted statistically different (p = 0.005).

In the four patients with possible IFD the median number (SFCs/10^6^ PBMCs with their 25th and 75th percentiles) of *Mucorales*-specific T cells producing IFN-γ was 40 (10–100); those T cells producing IL-10 was 80 (0–185); those T cells producing IL-4 was 80 (68–180) (Fig 4B).

When we compared the patients without IFD (group 1) with the two patients with proven IM, it resulted that the magnitude of specific immune responses, evaluated either as the number of samples with more than 100 SFCs or as median SFCs, was significantly greater in proven patients. In particular, the number of analysis with ≥ 100 SFCs/10^6^ PBMCs was 10 (7.41%) out of 135 in the first group and 8 (66.6%) out of 12 in the proven patients, respectively (p < 0.001). Moreover, these latter patients showed also significantly higher number of *Mucorales*-specific T cells producing IL-4 and IL-10 than the 15 patients without IFD (232 vs 0, p = 0.01; 43.5 vs 0, p = 0.03), suggesting an immune response polarized in a non-protective fashion versus fungi of the order Mucorales (Fig 2).

To verify the hypothesis that *Mucorales*-specific T cells, and, in particular, those producing type-2 cytokines, were associated with IM, we calculated the proportion of patients correctly "diagnosed" by the ELISpot assay and performed the receiver-operating characteristic (ROC) analysis, between the cases with a defined diagnosis with or without *Mucorales*-specific T cells, namely 17 patients versus 167 patients (Fig 1). The sensitivity and specificity of the test resulted 100% and 92%, respectively, when all the three types of *Mucorales*-specific T cells were considered, and 100% and 94.5% when only *Mucorales*-specific T cells producing IL-10 and IL-4 were evaluated (S1 Table and S2 Table). These data suggested that *Mucorales*-specific T cells producing type 2 cytokine were more suitable to detect IM and were consistent with what observed in the three proven IM cases previously reported [20].

The following cut-offs were derived: 30 SFCs/10^6^ PBMCs for *Mucorales*-specific T cells producing IL-10; 70 SFCs/10^6^ PBMCs for *Mucorales*-specific T cells producing IL-4 and 30 SFCs/10^6^ PBMCs when both *Mucorales*-specific T cells producing IL-10 and IL-4 were considered (S1 Fig).

To evaluate whether the 4 positive possible IFD could be upgraded to a higher degree of suspicion for IM, we analysed the sample positivity rates in these patients and made the comparison with patients with a proven diagnosis, by applying the derived cut-offs. When *Mucorales*-specific T cells producing IL-10 or IL-4 or both in combination were considered, the rates of positive samples in the 4 patients with possible IFD resulted 45% (5 out of 11) for each analysis. Such rates were significantly higher than those obtained in the 15 patients without IFD, namely 14% (5 out of 36) (p = 0.04), 8% (3 out of 36) (p = 0.01), 5.5% (2 out of 36) (p = 0.004), respectively, while did not differ from those obtained in the 2 patients with proven IM, 100% (4 out of 4) (p = 0.10, 0.10 and 0.27, respectively) (S3 Table).

In the 4 possible IFD patients, the occurrence of *Mucorales*-specific T cells preceded the detection of the pulmonary lesions in 2 out of 4 patients, namely patients n° 17 and 19; was contemporary in patient 18 and was demonstrated 4 days later in patient 16 (Table 2).

Of note, none of the patients with proven/probable IA, fusariosis and candidiasis showed the occurrence of *Mucorales*-specific T cells. Accordingly, neither the two patients with proven IM nor the 4 possible IFD patients, with *Mucorales*-specific T cells, demonstrated the occurrence of Aspergillus-specific T cells in all the tested samples.

4 (25%) out of the 16 possible IFD patients without *Mucorales*-specific T cells demonstrated the occurrence of Aspergillus-specific T cells in one or more samples, but only three showed such cells directed to more than one antigen or polarized in the production of type-2 cytokine, suggesting that such patients could be upgraded to a higher degree of suspicion for IA. The remaining subject demonstrated Aspergillus-specific immune responses similar to those reported in healthy donors [19, 24] (S4 Table).

A portion of the samples was not informative for all the cytokines studied: 19% (38/201) at T1, 5.5% (11/199) at T2, and 5% (10/198) at T3.

## Discussion

We have previously demonstrated the presence of *Mucorales*-specific T cells in three patients with documented IM [20]. Herein, we describe for the first time–in a prospective study–the frequency of *Mucorales*-specific T cells producing IFN-γ, IL-10 and IL-4 in a large cohort of hematologic patients at high risk for IFD. *Mucorales*-specific T cells could be demonstrated in 10.3% of 204 patients. Based on their characteristics, patients with positive results could be divided in two groups: group 1 (n = 15), unlikely to have IFD, and group 2 (n = 6), having proven IM or possible IFD, according to revised EORTC/MSG criteria [23].

In group 1, *Mucorales*-specific immune responses were polarized in a protective manner versus fungi of the order *Mucorales*, with a median number of *Mucorales*-specific T cells producing IFN-γ that was significantly higher than the number of specific T cells producing either IL-10 or IL-4 (p = 0.001). Similarly, in the setting of IA, healthy subjects display higher frequencies of specific T cells producing IFN-γ to *Aspergillus* antigens when compared to IA patients [19, 24–26].

Patients unlikely to have IFD and the 2 patients with proven IM were significantly different in: 1) their magnitude of specific immune responses, with the strongest responses in group 2 (p < 0.001) and 2) their frequencies of *Mucorales*-specific T cells producing IL-10 and IL-4, which were significantly higher in group 2 (p = < 0.03 and < 0.01, respectively). These findings suggest that group 2 patients had a stronger stimulation of the specific immune responses and such responses were shaped in a non-protective manner versus fungi of the order *Mucorales*. Both features are usually associated with high antigen loads [26, 27].

These data are consistent with previous reports: IM patients, as well as IA patients, have specific immune responses predominantly polarized to the production of IL-10 and/or IL-4 at the onset of the infection [19, 20, 22]. The increase of protective T cells occurs later and is associated with a favorable outcome [19].

Of note, the predominant type 2 cytokine secretion profile of *Mucorales*-specific T cells in the four patients with possible IFD in group 2, is similar to that of the two patients with proven IM in this study and to that of the three patients with proven IM previously reported [20], while significantly differed from the 15 positive patients without IFD.

These findings are important because most cases of IM are diagnosed as possible IFD, mainly due to the absence of sensitive and specific microbiological diagnostic tools and to the fact that tissue diagnosis, the gold standard, is only rarely performed in high risk patients, due to their several comorbidities [4, 11–13].

Of note, only three of the 16 remaining possible patients, without *Mucorales*-specific T cells, showed *Aspergillus*-specific T cells suggesting an increase likelihood of IA. This is not surprising as the whole cohort of patients have been extensively screened with the current cultural and non-cultural diagnostic methods (NCBDM) for the diagnosis of IA. Thus, it would have been very unlikely to upgrade a higher number of possible IFD patients to a higher degree of IA suspicion. Indeed, even if we hypothesize that the three possible IFD patients with *Aspergillus*-specific T cells are true IA cases, it should be acknowledged that such a common work-out correctly classified as IA 33 out of 36 (92%), with rates of infection matching those of the most recent epidemiological studies [8, 28, 29]. On the other hand, only 2 proven IM cases (0,9%) have been identified. Such rates are sensibly lower than the ones reported for IM in the same epidemiological studies [8, 28, 29]. These findings additionally emphasize the need for NCBDM for IM more than IA.

Our results further support the hypothesis that a type 2 cytokine profile is the immunological signature of IM. The concomitant absence of clinical signs of IFD in patients in the first group, characterized by a predominant type 1 cytokine profile, supports such a suggestion.

Some limitations of this study should be discussed. First, the samples taken at T1, usually a period of low leukocyte/lymphocyte counts, demonstrated the absence of vital T cells in approximately 20% of cases, thus showing not informative results. This finding may diminish the usefulness of the assay at least in the first part of post-treatment aplastic phase. However, this drawback can be overcome by repeating the analysis or by analyzing fresh blood samples without thawing, which is a procedure that further reduces the number of viable cells (unpublished data). Secondly, the study was not designed to determine the diagnostic accuracy of detecting *Mucorales*-specific T cells for the diagnosis of IM. It should be recognized that designing such a rigorous study may represent a problem, considering the tissue-based diagnosis difficulties previously mentioned [11]. However, if we use the ELISpot assay to upgrade at a higher level of IM suspicion the 4 cases of possible IFD, the assay show positivity rates, which seem to match the incidence of IM reported in several cohort of hematologic patients [29–31]. On the other hand, given the high rates of negative patients, our assay could be of help at least to reduce the number of patients at high risk for IM undergoing high dose antifungal treatment based only on clinical clues.

In conclusion, *Mucorales*-specific T cells may be routinely monitored in hematologic patients during chemotherapy/immunosuppressive regimens and alloSCT. The identification of high numbers of T cells producing IL-10 and/or IL-4 is associated with the diagnosis of proven IM. The detection of *Mucorales*-specific T cells should be explored as a NCBDM for the diagnosis of IM.

## Materials and Methods

### Patients

From October 2011 to January 2014, 4 tertiary care centers prospectively enrolled patients with acute myeloid or lymphoblastic leukemia undergoing induction chemotherapy, patients with other hematologic malignancies requiring either high dose chemotherapy or treatment with antithymocyte globulin, and patients undergoing allogeneic stem cell transplantation (alloSCT) into the study.

The study has been approved by the University of Modena and Reggio Emilia, Comitato Etico Provinciale di Modena (CE–protocol number 2414-63/11). Written informed consent was obtained from each patient, or from the next of kin, caretaker, or guardian on behalf of the minors enrolled in the study, according to the Declaration of Helsinki.

A sample of 30 mL of PB was drawn from patients at three pre-defined time-periods during their routine clinical examinations: T1 from day 15 to 30, T2 from day 30 to day 45, T3 from day 45 to 60, being T0 the start of chemotherapy or transplant procedure.

All the patients underwent a protocol-defined diagnostic work-up to define, when possible, the site and the etiology of the infectious/inflammatory process. The diagnostic work-up included cultures of blood, urine and feces specimens, molecular and serologic assays for diagnosing the most common viral pathogens, pulmonary and/or abdominal computed tomography (CT) scan, bronchoalveolar lavage (BAL) and, whenever feasible, biopsy of the affected tissue. BAL fluid was cultured for bacterial and fungal pathogens, mycobacteria, and Legionella, and was studied with immunofluorescent staining for Pneumocystis ***jiroveci***, and with molecular and cultural analysis for viral pathogens. The galactomannan assay (Platelia) was performed twice a week on serum samples of acute leukemia patients and alloSCT recipients, and, at discretion of the attending physician, in all the other patients. When available, it was also tested in BAL. An index ≥0.5 and ≥1.0 were considered positive, for serum and BAL respectively. Patients were also surveyed for the development of sinonasal and cerebral infections. When they developed symptoms suggestive of such infections, brain CT scan and/or magnetic resonance imaging scans were obtained. Whenever possible autopsy was performed and specimens of organs involved by the infectious process were sent for histopathology and culture. Hematoxylin-eosine, periodic acid-Schiff, Grocott or Gomori methenamine silver stains were all used to detect hyphal invasion in tissue samples.

The EORTC-MSG criteria have been used to define IFD cases [23].

### ELISpot Assay

Mucorales-specific T cells producing IFN-γ, IL-10 and IL-4 have been investigated through the Enzyme Linked ImmunoSpot (ELISpot) assay. The assay has been performed by three of the authors (DV, PB and GR), unaware of the clinical conditions of the patients, as previously described [19, 20, 22]. Briefly, PBMCs were co-cultured with specific antigens on 96-well flat bottom plates coated with anti-cytokine antibodies, namely anti-IFN-γ, IL-10 and IL-4, respectively (Mabtech AB, Nacka Strand, Sweden) [20].

The antigen preparation used was represented by heat-killed germinated conidia after four cycles of sonication at 280 W with a frequency of 24 KhZ, from fungi of the order *Mucorales*, at a concentration of 200,000 conidia/mL. All test conditions were carried out in duplicate or triplicate when possible. As previously reported, a sample was considered positive when all the following conditions were satisfied: 1) the presence of at least 5 SFCs; 2) the presence of more than 5 SFCs compared with the negative control; 3) the presence of a stimulation index of ≥2 (defined as ratio of SFCs in the sample versus the negative control). A sample was considered not informative when this last condition was not met [19, 20, 22]. Results have been reported as median number of SFCs/10^6^ PBMCs with their 25th and 75th percentiles.

*Aspergillus*-specific T cells producing IFN-γ, IL-10 and IL-4 have been detected as previously reported [19]. The antigens used were the seven *Aspergillus* recombinant antigens previously reported plus the protein of the family of β-(1,3)-glucan-modifying enzymes, namely SUN1p [19].

### Statistical Analysis

Median test was used to compare the median number of *Mucorales*-specific T cells producing IFN-γ-, IL-10 and IL-4, respectively, in positive patients, including those without IFD and those with proven IM.

Absolute numbers and percentages of the tests with ≥ 100 SFCs/10^6^ PBMCs were determined and compared between the two groups of patients, by using the Fisher's exact test. In the same groups, the differences in the frequencies of *Mucorales*-specific T cells producing IFN-γ-, IL-10- and IL-4, respectively, have been evaluated.

To define the cut-off values allowing to discriminate between the "true positive" cases from the “false positive” cases, sensitivity, specificity, positive and negative predictive values were calculated and ROC analysis was performed between the cases with a defined diagnosis, with or without *Mucorales*-specific T cells. The first positive or negative sample has been considered for the analyses. Fisher's exact test has been used to compare the rate of positive samples, according to the identified cut-offs, between the groups.

P values < 0.05 were considered statistically significant. Results were analyzed using STATA software (11.0, College Station, Texas, USA).

## Supporting Information

S1 Fig Receiver-operating characteristic (ROC) analyses to derive cut-offs. (DOCX)

S1 Table Proportion of patients correctly "diagnosed" by the ELISpot assay when all the three types of *Mucorales*-specific T cells were considered. (DOCX)

S2 Table Proportion of patients correctly "diagnosed" by the ELISpot assay when *Mucorales*-specific T cells producing IL-10 and IL-4 were considered. (DOCX)

S3 Table ELISpot results for *Mucorales*-specific T cells in the 21 positive patients. (DOCX)

S4 Table ELISpot results for *Aspergillus*-specific T cells in the group of 16 possible IFD cases negative for the presence of *Mucorales*-specific T cells. (DOCX)
