# Supplementary material for: Mucorales-Specific T Cells in Patients with Hematologic Malignancies
Source: PLoS One. 2016 Feb 12;11(2):e0149108. doi: 10.1371/journal.pone.0149108 (PMC4752352; doi:10.1371/journal.pone.0149108)
Supplement: S1 Fig — (DOCX) [file pone.0149108.s001.docx]

**S1 Fig. Receiver-operating characteristic (ROC) analyses to derive cut-offs.**

**
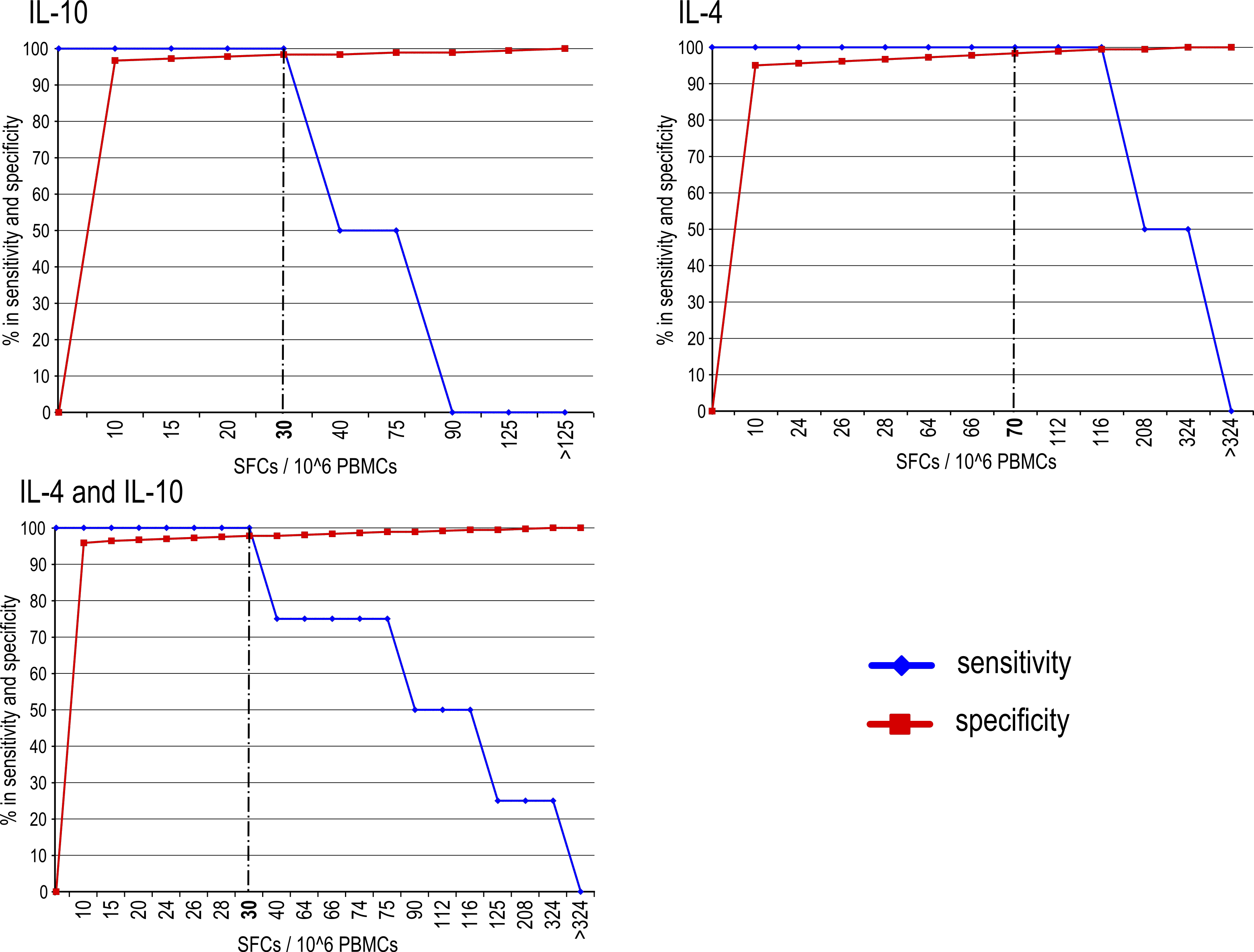
**

The ROC analyses were performed between the cases with a defined diagnosis, with or without *Mucorales*-specific T cells. The first positive or negative sample has been considered for the analyses. (A) ROC analysis of patients with IM versus those without, according to the presence of *Mucorales*-specific T cells producing IL-10. aROC = 0.98 (95% confidence interval: 0.95-0.99); optimal criterion ≥ 30; sensitivity: 1.00; specificity: 0.98; + LR: 60.6; - LR: 0.00. (B) ROC analysis of patients with IM versus those without according to the presence of *Mucorales*-specific T cells producing IL-4. aROC = 0.99 (95% confidence interval: 0.97-1.00); optimal criterion ≥ 70; sensitivity: 1.00; specificity: 0.98; + LR: 45.5; - LR: 0.00. (C) ROC analysis of patients with IM versus those without according to the presence of *Mucorales*-specific T cells producing IL-10 and IL-4. aROC = 0.998 (95% confidence interval: 0.97-1.00); optimal criterion ≥ 30; sensitivity: 1; specificity: 0.97; + LR: 45.2; - LR: 0.00.

aROC = area under the ROC curve; +LR = positive likelihood ratio; -LR = negative likelihood ratio.
