# Supplementary material for: Mucorales-Specific T Cells in Patients with Hematologic Malignancies
Source: PLoS One. 2016 Feb 12;11(2):e0149108. doi: 10.1371/journal.pone.0149108 (PMC4752352; doi:10.1371/journal.pone.0149108)
Supplement: S1 Table — (DOCX) [file pone.0149108.s002.docx]

**S1 Table. Proportion of patients correctly "diagnosed" by the ELISpot assay when all the three types of *Mucorales*-specific T cells were considered.**

|  | Patients with IM | Patients without IM |  |
| --- | --- | --- | --- |
| ELISpot positive | 2 | 15 | 17 |
| ELISpot negative | 0 | 167 | 167 |
|  | 2 | 182 |  |

Sensitivity = 2/2 = 100%

Specificity = 167/182 = 92%

Positive Predictive Value = 2/17 = 12%

Negative Predictive Value = 167/167 = 100%

IM = invasive mucormycosis; ELISpot = enzyme linked immunospot assay.
