# Supplementary material for: Mucorales-Specific T Cells in Patients with Hematologic Malignancies
Source: PLoS One. 2016 Feb 12;11(2):e0149108. doi: 10.1371/journal.pone.0149108 (PMC4752352; doi:10.1371/journal.pone.0149108)
Supplement: S2 Table — (DOCX) [file pone.0149108.s003.docx]

**S2 Table. Proportion of patients correctly "diagnosed" by the ELISpot assay when *Mucorales*-specific T cells producing IL-10 and IL-4 were considered.**

|  | Patients with IM | Patients without IM |  |
| --- | --- | --- | --- |
| ELISpot positive | 2 | 10 | 12 |
| ELISpot negative | 0 | 172 | 172 |
|  | 2 | 182 |  |

Sensitivity = 2/2 = 100%

Specificity = 172/182 = 94.5%

Positive Predictive Value = 2/12 = 16.6%

Negative Predictive Value = 172/172 = 100%

IM = invasive mucormycosis; ELISpot = enzyme linked immunospot assay.
