# Supplementary material for: Mucorales-Specific T Cells in Patients with Hematologic Malignancies
Source: PLoS One. 2016 Feb 12;11(2):e0149108. doi: 10.1371/journal.pone.0149108 (PMC4752352; doi:10.1371/journal.pone.0149108)
Supplement: S3 Table — (DOCX) [file pone.0149108.s004.docx]

**S3 Table. ELISpot results for *Mucorales-*specific T cells in the 21 positive patients.**

| **Pt n°** | **Time-points** | **Mucorales-specific T cells producing** | | |
| --- | --- | --- | --- | --- |
|  |  | **IFN-γ** | **IL-10** | **IL-4** |
| 1 | t1 | 0 | 0 | 0 |
|  | t2 | 65 | 0 | 0 |
|  | t3 | 35 | 0 | 0 |
| 2 | t2 | 110 | 10 | 0 |
|  | t3 | 115 | 10 | 0 |
| 3 | t1 | 0 | 0 | 0 |
|  | t2 | 90 | 0 | 0 |
|  | t3 | 30 | 12 | 0 |
| 4 | t1 | 0 | 0 | 0 |
|  | t2 | 0 | 0 | 0 |
|  | t3 | 65 | 0 | 0 |
| 5 | t3 | 325 | 0 | **112** |
| 6 | t1 | 0 | n.i. | n.i. |
|  | t2 | 0 | n.i. | 0 |
|  | t3 | 55 | 0 | 28 |
| 7 | t1 | 0 | n.i. | n.i. |
|  | t2 | 0 | 0 | 0 |
|  | t3 | 50 | 0 | 0 |
| 8 | t1 | 15 | **125** | 26 |
|  | t2 | 0 | 5 | 2 |
|  | t3 | 0 | 0 | 0 |
| 9 | t1 | 0 | 0 | 0 |
|  | t2 | 0 | 0 | 0 |
|  | t3 | 135 | **65** | 22 |
| 10 | t2 | 60 | 15 | 10 |
|  | t3 | 45 | **60** | 4 |
| 11 | t1 | 0 | 0 | 0 |
|  | t2 | 65 | 20 | 24 |
|  | t3 | 0 | 0 | 0 |
| 12 | t2 | 40 | **40** | **74** |
| 13 | t1 | 195 | n.i. | 66 |
| 14 | t1 | 40 | **90** | 64 |
|  | t2 | 0 | 0 | 0 |
|  | t3 | 0 | n.i. | n.i.. |
| 15 | t2 | 915 | n.i. | **208** |
|  | t3 | 640 | n.i. | n.i. |
| 16 | t1 | 0 | n.i. | n.i. |
|  | t2 | 240 | **185** | **80** |
|  | t3 | 100 | 0 | 60 |
| 17 | t2 | 25 | **60** | **76** |
|  | t3 | 0 | 0 | 0 |
| 18 | t1 | 10 | 0 | **146** |
|  | t2 | 0 | 0 | 0 |
|  | t3 | 10 | **80** | 68 |
| 19 | t1 | 40 | **435** | **180** |
|  | t2 | 65 | **140** | **180** |
|  | t3 | 0 | 0 | 0 |
| 20 | t2 | 125 | **75** | **324** |
|  | t3 | 120 | **57** | **386** |
| 21 | t2 | 140 | **30** | **116** |
|  | t3 | 275 | **30** | **140** |

Frequencies of Mucorales-specific T cells are reported as number of SFCs/10^6^ PBMCs. IFN-γ = interferon-gamma; IL-10 = interleukin-10; IL-4 = Pt = patient; t = time point; n.i. = not informative analysis. SFCs = spot forming cells. PBMCs = peripheral blood mononuclear cells. Results in bold indicate positive sample according with the derived cut-off.
