# Supplementary material for: Mucorales-Specific T Cells in Patients with Hematologic Malignancies
Source: PLoS One. 2016 Feb 12;11(2):e0149108. doi: 10.1371/journal.pone.0149108 (PMC4752352; doi:10.1371/journal.pone.0149108)
Supplement: S4 Table — (DOCX) [file pone.0149108.s005.docx]

**S4 Table. ELISpot results for *Aspergillus*-specific T cells in the group of 16 possible IFD cases negative for the presence of *Mucorales*-specific T cells.**

| **Pt n°** | **Time points** | ***Aspergillus*-specific T cells** | | |
| --- | --- | --- | --- | --- |
|  |  | **IFN-γ** | **IL10** | **IL4** |
| 22 | t1 | 8 | 0 | 0 |
|  | t2 | 0 | 8 | 6 |
|  | t3 | 0 | 4 | 0 |
| 23 | t1 | **145 CRF1p, 340 SUN1** | **270 CRF1p, 375 SUN1** | **114 CRF1p and SUN1** |
|  | t2 | **430 CRF1p, 540 SUN1** | **960 CRF1p, 15 GEL1, 55 alfa-glucosidase** | **320 CRF1p, 244 SUN1** |
|  | t3 | **345 CRF1p, 645 SUN1** | **270 CRF1p, 280 GEL** | **86 CRF1p, 210 SUN1, 42 GEL1** |
|  | t4 | **45 CRF1p, 35 GEL1, 45 SOD1, 30 beta-glucan, 255 SUN1** | **140 CRF1p, 650 SUN1** | **188 SUN1, 24 CRF1, 12 GEL1** |
| 24 | t1 | 5 | 0 | 4 |
|  | t2 | 0 | 0 | 0 |
|  | t3 | 0 | 0 | 0 |
| 25 | t1 | n.i. | 0 | 0 |
|  | t2 | n.i. | n.i. | n.i. |
|  | t3 | n.i. | n.i. | n.i. |
|  | t4 | n.i. | n.i. | n.i. |
| 26 | t1 | 0 | 0 | 0 |
|  | t2 | 0 | n.i. | 0 |
|  | t3 | 0 | n.i. | n.i. |
| 27 | t1 | n.i. | n.i. | n.i. |
|  | t2 | n.i. | n.i. | n.a |
|  | t3 | n.i. | n.i. | 0 |
| 28 | t1 | 0 | 0 | 0 |
|  | t2 | n.i. | n.i. | n.i. |
|  | t3 | 0 | n.i. | n.i. |
| 29 | t1 | **895 CRF1p** | 0 | 0 |
|  | t2 | 0 | n.i. | n.i. |
|  | t3 | 0 | n.i. | n.i. |
| 30 | t1 | 0 | 0 | 0 |
|  | t2 | n.i. | n.i. | n.i. |
|  | t3 | 0 | 0 | 0 |
| 31 | t1 | 0 | 0 | 0 |
|  | t2 | 0 | 0 | 0 |
| 32 | t1 | 0 | n.i. | n.a. |
| 33 | t1 | 0 | n.i. | n.a. |
|  | t2 | 0 | 0 | n.a. |
|  | t3 | 0 | 0 | 0 |
| 34 | t1 | 0 | 0 | 0 |
|  | t2 | 0 | 0 | 0 |
| 35 | t1 | 0 | 0 | 0 |
|  | t2 | 0 | 0 | 0 |
| 36 | t1 | **100 PEP1** | **40 CRF1p, 25 PEP1** | 0 |
|  | t2 | 0 | n.i. | 4 |
| 37 | t1 | **45 GEL1** | n.i. | n.i. |
|  | t2 | **195 SUN1** | n.i. | n.i. |
|  | t3 | **275 GEL1** | n.i. | n.i. |

CRF1p: ortholog of Crh1p associated in β1,6 glucan-chitin linkages (CRF1p) in S*accharomyces cerevisiae*; PEP1: aspartic protease; SUN1: protein of the family of β-(1,3)-Glucan-modifying Enzymes; GEL1: 1,3-β glucanosyltransferase; SOD1: superoxide dismutase. Results are expressed as number of Spot Forming Cells (SFCs)/10^6^ peripheral blood mononuclear cells to each antigen. n.i.: not informative for the absence of vital T cells. n.a.: not available.
